# Association between Self-Reported General and Mental Health and Adverse Outcomes: A Retrospective Cohort Study of 19 625 Scottish Adults

**DOI:** 10.1371/journal.pone.0093857

**Published:** 2014-04-04

**Authors:** Zia Ul-Haq, Daniel F. Mackay, Jill P. Pell

**Affiliations:** 1 Institute of Health & Wellbeing, Public Health, University of Glasgow, Glasgow, United Kingdom; 2 Institute of Public Health & Social Sciences, Khyber Medical University, KPK, Peshawar, Pakistan; National Center of Neurology and Psychiatry, Japan

## Abstract

**Objective:**

Self-reported general health and mental health are independent predictors of all-cause mortality. This study examines whether they are also independent predictors of incident cancer, coronary heart disease and psychiatric hospitalisation.

**Methods:**

We conducted a retrospective, population cohort study by linking the 19 625 Scottish adults who participated in the Scottish Health Surveys 1995–2003, to hospital admissions, cancer registration and death certificate records. We conducted Cox proportional hazard models adjusting for potential confounders including age, sex, socioeconomic status, alcohol, smoking status, body mass index, hypertension and diabetes.

**Results:**

Poor general health was reported by 1215 (6.2%) participants and was associated with cancer registrations (adjusted Hazard Ratio [HR] 1.30, 95% CI 1.10, 1.55), coronary heart disease events (adjusted HR 2.30, 95% CI 1.86, 2.84) and psychiatric hospitalisations (adjusted HR 2.42, 95% CI 1.65, 3.56). There was evidence of dose relationships and the associations remained significant after adjustment for mental health. 3172 (16%) participants had poor mental health (GHQ ≥4). After adjustment for general health, the associations between poor mental health and coronary heart disease events (adjusted HR 1.36, 95% CI 1.13, 1.63) and all-cause death (adjusted HR 1.38, 95% CI 1.23, 1.55) became non-significant, but mental health remained associated with psychiatric hospitalisations (fully adjusted HR 2.02, 95% CI 1.48, 2.75).

**Conclusion:**

Self-reported general health is a significant predictor of a range of clinical outcomes independent of mental health. The association between mental health and non-psychiatric outcomes is mediated by general health but it is an independent predictor of psychiatric outcome. Individuals with poor general health or mental health warrant close attention.

## Introduction

Self-reported general health (GH) is a simple but reliable measure of overall, subjective well-being [1], [2]. Several studies have demonstrated that it is a significant predictor of all-cause mortality [3]–[5]. There is a relative paucity of studies on the association between GH and specific diseases, especially in relation to non-fatal outcomes such as hospitalisation. Also, it is not clear to what extent the association is independent of lifestyle factors [6], [7]. Studies suggest that GH is influenced more by physical health than mental health (MH) [8], [9]. Therefore, it is unclear whether GH predicts psychiatric hospitalisations as well as physical disease.

The General Health Questionnaire-12 (GHQ) is a validated measure of MH and has been widely used in population studies [10]. GHQ score is predictive of both all-cause mortality [11]–[13] and cardiovascular disease (CVD) [14], [15]. It is not clear whether the association is independent of GH. Higher GHQ scores, indicative of poor MH, have also been observed among patients with other chronic diseases such as cancer in cross-sectional studies [16], but the association has rarely been studied longitudinally. Relatively few studies have formally tested for interactions but those that have suggest that the association between poor GH and all-cause death is greater in men than women, whilst the association between poor MH and all-cause death is stronger in women than men [17], [18].

We previously demonstrated, in a cross-sectional study of more than 160 000 UK Biobank participants, that the association between unhappiness and adiposity was no longer apparent after adjusting for GH [19]. In contrast, the association between GH and adiposity remained significant even after adjustment for unhappiness. Based on these findings, we hypothesized that GH may be an independent predictor of other forms of morbidity and that any association between MH and physical morbidity will be explained by GH. Some other recent studies have made similar hypotheses but they have rarely been tested, particularly in longitudinal studies [20]. It is possible that they have a direct causal effect. Perceived health (general or mental) may impact on lifestyle and therefore incident disease and survival. It is also possible that perceived health is a marker of sub-clinical morbidity or that the incident diseases and perceived health share common risk factors. The aim of this study was to determine whether GH and MH were independent predictors of incident cancer, psychiatric and coronary heart disease as well as all-cause deaths, and whether the associations varied by sex.

## Methods

### Data sources

The Scottish Health Survey (SHS) collects information on health and health-related risk factors in the Scottish general population (http://www.scotland.gov.uk/Topics/Statistics/Browse/Health/scottish-health-survey). The first SHS was undertaken in 1995. It was repeated in 1998 and 2003 and, since 2008, has been undertaken annually. Different households have been targeted in each survey and the recruitment rates have exceeded 60%. The detailed methodology of the surveys has been explained elsewhere [21], [22]. Briefly, the members of participating households are interviewed by trained data collectors who use computer assisted personal interviewing (CAPI) to collect information on demographics (including age, sex, postcode of residence and social class), lifestyle (including smoking status and alcohol consumption) and self-reported health. During a subsequent visit, a nurse uses standard operating procedures and calibrated tools to record height, weight and blood pressure. More than 90% of SHS participants have consented to passive follow up via record linkage to routine administrative data. The SHS records have been linked, at an individual level, to several Scotland-wide databases including: death certificates (collated by the General Registrar Office), admissions to acute hospitals (Scottish Morbidity Record SMR01), psychiatric hospitalisation (Scottish Morbidity Record SMR04) and cancer registrations (Scottish Morbidity Record SMR06) [23]. Cancer was defined by the International Classification of Diseases-10 (ICD-10) codes C00-C97. Psychiatric hospitalisation was defined as first hospitalisation with an ICD-10 code F00-F99 in principal position. Coronary heart disease event was defined as death or hospitalisation due to coronary heart disease. The latter was defined as first hospitalisation with an ICD-10 code I20-I25 in the principal position. The SMR data are reported to be 94% accurate and 99% complete [24]. The extract of linked data provided follow-up to a censor date of 31 December 2011.

### Inclusion criteria and definitions

For this study, we combined the first three SHS surveys (1995, 1998 and 2003) and restricted our analyses to participants aged ≥16 years. We excluded participants with established cancer, coronary heart disease or psychiatric disease at recruitment. Age was categorised into 16–29, 30–44, 45–59 and ≥60 years. Body mass index was categorised into underweight (<18.5 kg/m^2^), normal weight (18.5–24.9 kg/m^2^), overweight (25.0–29.9 kg/m^2^), class I (30.0–34.9 kg/m^2^), class II (35.0–39.9 kg/m^2^) and class III obese (≥40 kg/m^2^). The occupation of the household's main income earner was used to derive social class: I (professional), II (managerial), III NM (skilled non manual), III M (skilled manual), IV (semi-skilled manual) and V (unskilled manual) [25], and area-based deprivation was derived from the postcode of residence using general population quintiles of the Scottish Index of Multiple deprivation (SIMD) (http://www.scotland.gov.uk/Topics/Statistics/SIMD). Smoking status and alcohol consumption were both self-reported and were categorised as never, previous and current, and never, previous, within limit and excessive respectively. Prevalent hypertension (blood pressure ≥140/90 mmHg or use of anti-hypertensive medication) and diabetes were based on self-report of a physician diagnosis. GH was self-reported and based on responses to the question; “In general, how would you rate your overall health.” Possible responses were: very good, good, fair, bad and very bad. In this study, the five responses were collapsed into three categories: good (very good or good), fair and bad (bad or very bad). MH was based on participants' responses to the GHQ-12 questionnaire. GHQ-12 scores range from 0 to 12, with 12 indicating the poorest mental health. In this study, the scores were collapsed into three categories: 0, 1–3 and ≥4.

### Statistical analyses

Differences in the characteristics of participants by GH and MH were analysed using chi-square tests for categorical data, and chi-square tests for trend for ordinal data. Separate Cox proportional hazard models were used to examine the association between GH and four separate outcomes: all-cause deaths, cancer registrations, coronary heart disease events (admissions or death) and psychiatric admissions. The model was run in three stages: adjusted for age only; adjusted for age, sex, social class, SIMD quintile, body mass index, alcohol consumption, smoking status, hypertension, diabetes and survey year; and adjusted for these covariates plus MH. We tested for statistical interactions between GH and the covariates, including sex. The same Cox proportional hazard models were then repeated for MH, adjusting for GH in the final model. All statistical analyses were performed using Stata version 12.1 (StataCorp, College Station, Texas). Statistical significance was defined as p<0.05.

## Results

Of the 21 252 survey participants, 1627 were excluded because of prevalent disease: 559 had cancer, 629 coronary heart disease and 439 psychiatric disease. Therefore, 19 625 were eligible for inclusion. Of these, 8858 (45.1%) were men, 6494 (33.1%) were current smokers, 4228 (21.5%) consumed excessive amounts of alcohol and 3919 (20.0%) had either hypertension, diabetes or both (Table 1). Their mean age at recruitment was 45 years (SD 16 years), and their mean body mass index was 26.7 kg/m^2^ (SD 5.0 kg/m^2^). Participants were followed-up for up to 17 years (mean 12 years), providing a total of 237,463 person years of follow-up. Incident events included 2077 all-cause deaths, 1777 cancer registrations, 829 coronary heart disease hospitalisations or deaths and 287 hospitalisations for psychiatric disease.

**Table 1:** Baseline characteristics of study population in relation to self-reported general health and mental health (measured by GHQ-12).

	Self-reported General Health				GHQ-12 score			
	Good	Fair	Bad		0	1–3	≥4	
	N = 14 741	N = 3669	N = 1215		N = 11 720	N = 4733	N = 3172	
	N (%)	N (%)	N (%)	P value	N (%)	N (%)	N (%)	P value
**Sex**								
Male	6630 (45.0)	1644 (44.8)	584 (48.1)	0.104	5574 (47.6)	2087 (44.1)	1197 (37.7)	<0.001
Female	8111 (55.0)	2025 (55.2)	631 (51.9)		6146 (52.4)	2646 (55.9)	1975 (62.3)	
**Age (years)**								
16–29	2990 (20.3)	504 (13.7)	59 (4.9)	<0.001	2062 (17.6)	966 (20.4)	525 (16.6)	0.001
30–44	5294 (35.9)	904 (24.6)	257 (21.2)		3845 (32.8)	1506 (31.8)	1104 (34.8)	
45–59	3947 (26.8)	1131 (30.8)	458 (37.7)		3253 (27.8)	1294 (27.3)	989 (31.2)	
≥60	2510 (17.0)	1130 (30.8)	441 (36.3)		2560 (21.8)	967 (20.4)	554 (17.5)	
**Body Mass Index**								
Underweight	179 (1.2)	73 (2.0)	31 (2.6)	<0.001	142 (1.2)	66 (1.4)	75 (2.4)	0.152
Normal-weight	6079 (41.2)	1185 (32.3)	360 (29.6)		4437 (37.9)	1913(40.4)	1274 (40.2)	
Overweight	5738 (38.9)	1308 (35.7)	411 (33.8)		4684 (40.0)	1704 (36.0)	1069 (33.7)	
Class I obese	2071 (14.1)	738 (20.1)	238 (19.6)		1803 (15.4)	761 (16.1)	483 (15.2)	
Class II obese	493 (3.3)	256 (7.0)	108 (8.9)		461 (3.9)	208 (4.4)	188 (5.9)	
Class III obese	181 (1.2)	109 (3.0)	67 (5.5)		193 (1.7)	81 (1.7)	83 (2.6)	
**SIMD**								
1 (Most deprived)	2582 (17.5)	1083 (29.5)	488 (40.2)	<0.001	2157 (18.4)	1073 (22.7)	923 (29.1)	<0.001
2	2969 (20.1)	911 (24.8)	331 (27.2)		2418 (20.6)	1023 (21.6)	770 (24.3)	
3	3150 (21.4)	736 (20.1)	184 (15.1)		2516 (21.5)	1000 (21.1)	554 (17.5)	
4	3075 (20.9)	574 (15.7)	136 (11.2)		2458 (21.0)	841 (17.8)	486 (15.3)	
5 (Least deprived)	2965 (20.1)	365 (10.1)	76 (6.3)		2171 (18.5)	796 (16.8)	439 (13.8)	
**Social Class**								
I & II	5111 (34.7)	807 (22.0)	201 (16.5)	<0.001	3713 (31.7)	1558 (32.9)	848 (26.7)	<0.001
III NM	2223 (15.1)	466 (12.7)	129 (10.6)		1654 (14.1)	664 (14.0)	500 (15.8)	
III M	4452 (30.2)	1297 (35.4)	472 (38.9)		3826 (32.7)	1413 (29.9)	982 (31.0)	
IV/V	2955 (20.1)	1099 (30.0)	413 (34.0)		2527 (21.6)	1098 (23.2)	842 (26.5)	
**Smoking status**								
Never	6994 (47.5)	1195 (32.6)	244 (20.1)	<0.001	5330 (45.5)	2021 (42.7)	1082 (34.1)	<0.001
Previous	3445 (23.4)	911 (24.8)	342 (28.2)		2904 (24.8)	1140 (24.1)	654 (20.6)	
Current	4302 (29.2)	1563 (42.6)	629 (51.8)		3486 (29.7)	1572 (33.2)	1436 (45.3)	
**Alcohol consumption**								
Never	650 (4.4)	218 (5.9)	101 (8.3)	<0.001	581 (5.0)	233 (4.9)	155 (4.9)	0.244
Previous	401 (2.7)	248 (6.8)	152 (12.5)		380 (3.2)	198 (4.2)	223 (7.0)	
Within limits	10421 (70.7)	2436 (66.4)	770 (63.4)		8291 (70.7)	3255 (68.8)	2081 (65.6)	
Excessive	3269 (22.2)	767 (20.9)	192 (15.8)		2468 (21.1)	1047 (22.1)	713 (22.5)	
**Medical comorbidity**								
No	12591 (85.4)	2467 (67.3)	648 (53.3)	<0.001	9637 (82.2)	3736 (78.9)	2333 (73.6)	<0.001
Yes	2150 (14.6)	1202 (32.8)	567 (46.7)		2083 (17.8)	997 (21.1)	839 (26.5)	
**GHQ-12 score**								
0	9930 (67.4)	1588 (43.3)	202 (16.6)	<0.001	-	-	-	
1–3	3285 (22.3)	1114 (30.4)	334 (27.5)		-	-	-	
≥4	1526 (10.4)	967 (26.4)	679 (55.9)		-	-	-	
**Self-reported general health**								
Good	-	-	-		9930 (84.7)	3285 (69.4)	1526 (48.1)	<0.001
Fair	-	-	-		1588 (13.6)	1114 (23.5)	967 (30.5)	
Bad	-	-	-		202 (1.7)	334 (7.1)	679 (21.4)	

*P* values were determined by χ2 test.GHQ General Health Questionnaire; N number; SIMD Scottish Index of Multiple Deprivation; NM non- manual; M manual.

Overall, 3669 (18.7%) participants classified themselves as being in fair GH and 1215 (6.2%) in bad GH. There was no significant difference between men and women. Compared to those with good GH, those with bad GH were more likely to be old, socioeconomically deprived, obese, smoke and have hypertension or diabetes, but were less likely to consume excessive amounts of alcohol (Table 1). Overall, 3172 (16.2%) participants had poor MH (GHQ-12 score ≥4). Compared to those with good MH (GHQ-12 score 0), they were more likely to be women, young, socioeconomically deprived, smoke, and have diabetes or hypertension, but were not significantly different in terms of alcohol consumption and body mass index (Table 1). Of the 1215 participants with bad GH, 202 (16.6%) had good MH (GHQ-12 score 0), and of the 3172 participants who had poor MH (GHQ-12 score ≥4), 1526 (48.1%) reported good GH.

**Figure 1:**
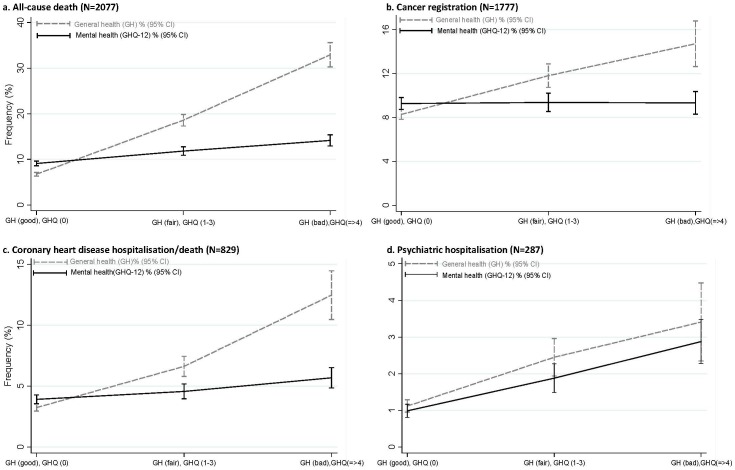
Frequency (%) of adverse outcomes by self-reported general health (GH) and mental health [GHQ-12 score].

There were positive dose relationships between baseline GH and all-cause deaths, cancer registrations, coronary heart disease events, and psychiatric hospitalisations (Figure 1). Compared with those with good GH, participants with bad GH were significantly more likely to experience all of the adverse outcomes when adjusted for age only (Table 2). Further adjustment, for all potential confounders, attenuated the associations slightly but they all remained statistically significant (Table 2). When also adjusted for MH, the associations with all-cause death, cancer registration and coronary heart disease hospitalisation or death changed very little and all remained statistically significant (Table 2). The magnitude of the association between GH and psychiatric admissions fell (from HR 2.42 to HR 1.73). Nonetheless, it remained statistically significant (Table 2). There was a significant interaction with age (p<0.001), but not with sex (p = 0.443). The subgroup analysis by age demonstrated that the association between bad GH and all-cause deaths were stronger in young adults, aged 16–29 years (HR 6.90), but it remained significant in other age-groups, 30–44 years (HR 3.75), 45–59 years (2.73) and ≥60 years (2.28) (Table S1).

**Table 2:** Cox proportional hazard models of the association between self-reported general health and adverse outcomes of Scottish adults (n = 19 625).

	Model 1		Model 2		Model 3	
	HR (95% CI)	P value	HR (95% CI)	P value	HR (95% CI)	P value
**All-cause death**						
Good	1.00		1.00		1.00	
Fair	2.10 (1.90, 2.31)	<0.001	1.67 (1.51, 1.86)	<0.001	1.66 (1.50, 1.85)	<0.001
Bad	3.74 (3.32,4.20)	<0.001	2.50 (2.20, 2.83)	<0.001	2.48 (2.16, 2.85)	<0.001
**Cancer registration**						
Good	1.00		1.00		1.00	
Fair	1.13 (1.01, 1.27)	0.032	1.08 (0.96, 1.22)	0.195	1.08 (0.96, 1.22)	0.189
Bad	1.41 (1.20, 1.66)	<0.001	1.30 (1.10, 1.55)	0.003	1.32 (1.09, 1.58)	0.003
**Coronary heart disease hospitalisation/death**						
Good	1.00		1.00		1.00	
Fair	1.75 (1.49, 2.06)	<0.001	1.39 (1.18, 1.64)	<0.001	1.37 (1.16, 1.63)	<0.001
Bad	3.34 (2.75, 4.06)	<0.001	2.30 (1.86, 2.84)	<0.001	2.26 (1.79, 2.84)	<0.001
**Psychiatric hospitalisation**						
Good	1.00		1.00		1.00	
Fair	2.37 (1.82, 3.09)	<0.001	1.88 (1.43, 2.47)	<0.001	1.60 (1.21, 2.13)	0.001
Bad	3.80 (2.65, 5.46)	<0.001	2.42 (1.65, 3.56)	<0.001	1.73 (1.15, 2.62)	0.009

HR, hazard ratio; CI, confidence interval

Model 1 adjusted for age; Model 2 adjusted for age, sex, social class, SIMD quintile, body mass index, alcohol consumption, smoking status, hypertension, diabetes and survey year; Model 3 adjusted for same covariates as Model 2 plus self-rated mental health (GHQ-12 score).

There were positive dose relationships between MH and all-cause death, coronary heart disease events and psychiatric hospitalisations, but not with cancer registrations (Figure 1). In the Cox proportional hazard model, poor MH was associated with a significantly increased risk of all-cause death and cardiovascular events (Table 3). After adjustment for potential confounders, the hazard ratios were attenuated but remained statistically significant. When also adjusted for GH, the associations became statistically non-significant. There was no significant association between MH and cancer registrations. MH was a significant, independent predictor of psychiatric admissions, and remained so after adjustment for GH (Table 3). There was no significant interaction with sex (p = 0.163).

**Table 3:** Cox proportional hazard models of the association between self-reported mental health (GHQ-12 score) and adverse outcomes of Scottish adults (n = 19 625).

	Model 1		Model 2		Model 3	
	HR (95% CI)	P value	HR (95% CI)	P value	HR (95% CI)	P value
**All-cause death**						
0	1.00		1.00		1.00	
1–3	1.33 (1.20, 1.47)	<0.001	1.25 (1.13, 1.39)	<0.001	1.06 (0.95, 1.18)	0.273
≥4	1.75 (1.57, 1.96)	<0.001	1.38 (1.23, 1.55)	<0.001	1.00 (0.88, 1.13)	0.968
**Cancer registration**						
0	1.00		1.00		1.00	
1–3	1.04 (0.93, 1.16)	0.515	1.04 (0.93, 1.16)	0.509	1.01 (0.90, 1.13)	0.883
≥4	1.09 (0.95, 1.24)	0.210	1.05 (0.92, 1.20)	0.468	0.97 (0.84, 1.13)	0.724
**Coronary heart disease hospitalisation/death**						
0	1.00		1.00		1.00	
1–3	1.20 (1.02, 1.42)	0.027	1.18 (1.00, 1.39)	0.055	1.05 (0.89, 1.24)	0.571
≥4	1.57 (1.32, 1.88)	<0.001	1.36 (1.13, 1.63)	0.001	1.04 (0.85, 1.27)	0.732
**Psychiatric hospitalisation**						
0	1.00		1.00		1.00	
1–3	1.88 (1.42, 2.49)	<0.001	1.76 (1.33, 2.33)	<0.001	1.61 (1.21, 2.14)	0.001
≥4	3.02 (2.28, 4.00)	<0.001	2.41 (1.81, 3.21)	<0.001	2.02 (1.48, 2.75)	<0.001

HR, hazard ratio; CI, confidence interval

Model 1 adjusted for age; Model 2 adjusted for age, sex, social class, SIMD quintile, body mass index, alcohol consumption, smoking status, hypertension, diabetes and survey year; Model 3 adjusted for same covariates as Model 2 plus self-rated general health.

## Discussion

GH was a significant predictor of incident cancer, psychiatric hospitalisations and coronary heart disease events as well as all-cause mortality. The associations were independent of potential confounders and MH, and there was evidence of a dose relationship. In contrast MH was not associated with cancer and the association with coronary heart disease and all-cause death was lost when adjusted for GH. However, it was a significant independent predictor of psychiatric hospitalisations. There were no statistically significant differences in the associations between men and women.

The United Kingdom “Annual Population Survey 2011/12” revealed that GH was the most important determinant of personal well-being; followed by employment and relationship status [26]. Those with bad GH were, on average, less satisfied, more unhappy and more likely to be depressed. In a previous cross-sectional study, using data from UK Biobank, we found a significant association between unhappiness and obesity, particularly among women. When adjusted for self-reported health, the association was no longer significant. In contrast, the association between GH and adiposity remained significant after adjustment for unhappiness [19].

Bad GH has been widely studied as a predictor of all-cause mortality. The latest meta-analysis collated data from 14 studies published up to 2003, and reported a pooled relative risk (RR) of 1.92 (95% CI 1.64, 2.25) [27]. Five of the 14 studies adjusted for baseline depression, reducing the pooled RR to 1.87 (95% CI 1.31, 2.67). None of the studies used the GHQ to measure MH. Our study corroborates the previous studies in demonstrating an association between bad GH and all-cause mortality (adjusted HR 2.50, 95% CI 2.20, 2.83, p<0.001), but adjustment for MH made very little difference to the magnitude of the association; the hazard ratio changing from 2.50 to 2.48. Most of the studies included in the meta-analysis were conducted in the USA. Only one of the studies was conducted in the United Kingdom; it did not adjust for mental health and included only participants aged 65 years and above [28]. Overall, the number of participants ranged from 630 to 6523, with a total of 31 350 participants. The maximum number of deaths in any study was 1919, with a total of 8437 deaths. Among the studies that adjusted for depression, the maximum number of participants in any study was 3971 and the maximum number of deaths was 854. Our study of almost 20 000 adults followed-up for up to 17 years provided information on more than 2000 deaths.

In contrast to all-cause mortality, GH has not been extensively studied as a predictor of other health outcomes. In a cohort study of 4770 USA adults aged 51–61 years, Latham and Peek demonstrated associations between GH and a number of self-reported incident conditions including arthritis, diabetes, lung disease, stroke [29]. Compared with participants who had poor GH, those with excellent GH were significantly less likely to report subsequent coronary heart disease (adjusted HR 0.80). In contrast, GH was not associated with cancer. Van der Linde and colleagues conducted a prospective cohort study of 20 041 residents of Norfolk, England, aged 39–74 years. Over an average follow-up of 11 years (range 0–14 years), they recorded 2176 cardiovascular disease events, and they reported a HR of 3.3 (95% CI 2.4, 4.4) for the association between bad GH and cardiovascular disease [20]. In our study, bad GH was associated with both coronary heart disease and cancer but the association with coronary heart disease (HR 2.30, 95% CI 1.86, 2.84, p<0.001) was stronger than with cancer (HR 1.30, 95% CI 1.10, 1.55, p = 0.003) and only the former demonstrated a dose-relationship. Very few studies have explored the relationship between GH and future depression. Kosloski et al showed a small but significant effect of GH on depressive symptoms over time in an autoregressive, cross-lagged panel study of 7475 US participants [30]. Badawi G et al analyzed repeated cross-sectional surveys of 1265 Canadian adults with type II diabetes [31]. When re-assessed after three years, those with poor GH at baseline had a twofold increased risk of self-reported depression even after controlling for potential confounders (adjusted odds ratio 2.05, 95% CI 1.20, 3.48). In a prospective cohort study of 1468 Canadians aged 65 years or older, St John and Montgomery reported that GH was associated with future dementia among participants who were cognitively intact at baseline [32]. Similar, findings were reported in a French prospective cohort study [33]. Both studies reported hazards ratios of around 2.5.

Some studies have reported that GH was a stronger predictor of health outcomes in men than women [34], whilst others have reported the reverse [35], and some have reported no difference [20], [28]. Most studies have not reported interaction tests and many were underpowered to do so. We formally tested for an interaction with sex but no significance difference was found. There was a significant interaction with age, such that the association between bad GH and all-cause deaths were stronger in young adults but it persists in all age groups.

Fewer studies have been conducted on MH than GH. A recent large study using multiple English Health Surveys reported a hazard ratio for all-cause death of 1.37 (95% CI 1.23, 1.51) for GHQ scores of 4–6, and a hazard ratio of 1.67 (95% CI 1.41, 2.00) for GHQ scores ≥7 [13]. In contrast, other studies have reported no significant associations in either men or women [18]. Our study demonstrated a dose relationship between MH and all cause mortality with significantly higher risk even among participants with GHQ-12 scores between 1 and 3 (adjusted HR 1.25, 95% CI 1.13, 1.39, p<0.001). Nicholson et al followed-up 5449 men aged 35–55 years who were living in London, over a mean of 6.8 years. They reported a significant association between poor MH and coronary heart disease events, which was highest among those with recent onset distress (HR 1.77, 95% CI 1.13, 2.78) [15]. Another study reported an odds ratio of 1.83 (95% CI 1.5, 2.3) for the association between psychological distress at baseline and self-reported coronary heart disease among 2383 middle-aged participants working in London [36]. Our finding of no association between MH and cancer is consistent with previous studies. A prospective cohort study of Scottish adults with 30 years follow-up reported a significant association between moderate stress (GHQ 4–5) and both breast cancer (HR 2.16, 95% CI 1.00, 4.71) and prostate cancer (HR 1.65, 95% CI 1.20, 2.27), but no significant association with more severe stress (≥6) [13]. A Scottish retrospective cohort study of 15 453 participants who were followed up for an average of 7 years, demonstrated a significant association with cancer mortality among the 295 participants who had a history of cancer at baseline (HR 1.97, 95% CI 1.05, 3.71), but not in cancer-free individuals [37].

Our study did not explore the underlying mechanism by which perceived health may impact on health and life-expectancy. It has been suggested that perceived health provides additional information by incorporating both subjective assumptions and objectively measured health [38]. Previous studies have suggested that personality traits, such as optimism, may inflate self-assessment of health [39]. Others have related good GH with better immune-responses [40]. GH may detect very early symptoms of underlying disease at subclinical stage. Finally, perceived health may reflect an individual's attitude towards improving health, and thus adopting preventive measures [4]. MH was not associated with the risk of all-cause mortality when adjusted for GH. It possibly reflects the fact that GH is a measure of overall perceived health and includes perceived MH, whereas MH is only one component.

Use of the Scottish Health Survey provided us with a large, representative sample of Scottish adults recruited from the general population, and enabled us to adjust for a series of potential confounders and test for interactions. Many previous studies have used binary variables for GH and MH. By using ordinal data we were able to examine whether there were evidence of a dose relationship. The GHQ-12 is validated measure of MH and has been widely used in epidemiological studies. The World Health Organization recommended that GH should be a standard component of health surveys and is, thus, worldwide, the most frequently used survey measure of health [2]. Use of a cohort, rather than cross-sectional, design enabled us to demonstrate a temporal relationship between baseline GH and MH and incident disease, and avoid reverse causation. The Scottish Morbidity Record system has Scotland wide coverage and is subjected to regular quality assurance checks. Linkage to SMR records and death certificates enabled us to study both all-cause mortality and disease-specific outcomes in the same cohort.

## Conclusions

GH is a strong predictor of all-cause death, cancer, coronary heart disease events and psychiatric hospitalisations. The association is independent of potential confounders and MH. Further research is required to understand the underlying mechanism. Individuals with poor GH are a higher risk group who may merit closer surveillance and earlier intervention. Perceived health is as important as objective measures of health risk, and should be considered in identifying how best to target public health and health care interventions.

## Supporting Information

Table S1 Cox proportional hazard models of the association between self-reported general health (GH) and all-cause mortality by age.
(DOCX)
