# Supplementary material for: Association between Self-Reported General and Mental Health and Adverse Outcomes: A Retrospective Cohort Study of 19 625 Scottish Adults
Source: PLoS One. 2014 Apr 4;9(4):e93857. doi: 10.1371/journal.pone.0093857 (PMC3976324; doi:10.1371/journal.pone.0093857)
Supplement: Table S1 — Cox proportional hazard models of the association between self-reported general health (GH) and all-cause mortality by age. (DOCX) [file pone.0093857.s001.docx]

**Table S1** Cox proportional hazard models of the association between self-reported general health (GH) and all-cause mortality by age ^a^

|  | **16-29 years** | | **30-44 years** | | **45-59 years** | | **≥60 years** | |
| --- | --- | --- | --- | --- | --- | --- | --- | --- |
|  | HR (95% CI) | P value | HR (95% CI) | P value | HR (95% CI) | P value | HR (95% CI) | P value |
| Good | 1.00 |  | 1.00 |  | 1.00 |  | 1.00 |  |
| Fair | 2.21 (0.98, 4.97) | 0.055 | 1.59 (1.09, 2.34) | 0.017 | 1.86 (1.54, 2.26) | <0.001 | 1.59 (1.40, 1.81) | <0.001 |
| Bad | 6.90 (1.92, 24.83) | 0.003 | 3.75 (2.36, 5.95) | <0.001 | 2.73 (2.17, 3.44) | <0.001 | 2.28 (1.94, 2.69) | <0.001 |

HR, hazard ratio; CI, confidence interval

^a^ Adjusted for sex, social class, SIMD quintile, body mass index, alcohol consumption, smoking status, hypertension, diabetes and survey year
